# Immune Thrombocytopenic Purpura and Hemolytic Anemia Secondary to Hepatitis A

**Published:** 2017-04-01

**Authors:** Ghasem Miri-Aliabad, Somayeh Rashidi

**Affiliations:** 1 Assistant Professor of Pediatric Hematology-Oncology, Children and Adolescent Health Research center, Zahedan University of Medical Sciences, Zahedan, Iran; 2 Pediatrician, Zahedan University of Medical Sciences, Zahedan, Iran

**Keywords:** Hepatitis A, Immune thrombocytopenic purpura, Hemolytic anemia

## Abstract

Hepatitis A is common in children and usually is a self-limiting disease. Although extrahepatic and hematological immune manifestations following acute hepatitis A virus (HAV) infection have rarely been reported, they are frequently observed in other viral hepatitis. In this paper, we report the case of a 3-year-old girl who developed immune thrombocytopenic purpura (ITP) and hemolytic anemia after HAV infection. She was presented with malaise, pallor, ecchymosis, petechiae and purpura on the trunk and extremities**.**

## Introduction

 Hematologic manifestations of hepatitis B and C are commonly seen, but thrombocytopenia and hemolytic anemia associated with hepatitis A are very rare, and most cases have been in adults and older children.^1^ Immune thrombocytopenia after hepatitis A may be due to antiplatelet antibodies, anti cardiolipin antibodies ornon-specific deposition of immune complexes on the surface of platelet.^2^ Although autoimmune hemolytic anemia occurs after hepatotropic viruses such as CMV, HBV, EBV, it rarely has been reported after HAV, and was first reported in a 46-year-old man by Tibble JA et al.^3^Hemophagocyticlymphohistiocytosis (HLH) syndrome and aplastic anemia have been also seen rarely during the course of hepatitis A infection. Though, other transient hematologic complications may appear too.^4^ Here, we introduce the case of a 3-year-old girl whose HAV infection had primarily been presented with immune hemolytic anemia and thrombocytopenic purpura.

**CASE PRESENTATION**

The patient is a 3-year-old girl presented with complaints of anorexia and ecchymosis. She was well-developed and well-nourished with no significant past medical history. On physical examination, mild scleral icterus, pallor, petechiae, purpura, ecchymosis, especially on the trunk and extremities, and hepatomegaly (3 cm below costal margin in the midclavicular line) were evident. Fever, lymphadenopathy, or splenomegaly were all absent. Other physical examinations indicated no abnormality. Initial laboratory evaluation of the patient was as follows: WBC: 14200/mm^3^, PMN: 48%, HB: 8.6g/dl, MCV: 82 fl, PLT: 14000 / mm^3^, Retic: 3.6 %, Bili T: 3.5 mg/dl, Bili D: 1.8 mg/dl, SGOT: 602 IU/L, SGPT: 617 IU/L, Alkaline phosphatase: 1814 IU/L, GGT: 122 IU/L.

Renal function tests, electrolytes, PT, PTT, ANA, anti-dsDNA, CH50, G6PD were normal. IgM antibody anti HAV was positive, but serologic studies for hepatitis B, C, EBV, CMV and HIV were normal. Direct anti-globin test (direct Coombs) was positive. Abdominal ultrasonography was normal other than hepatomegaly. Peripheral blood smear showed polychromasia, giant platelets and increase in the number of atypical lymphocytes. Bone marrow smear revealed megakaryocytic hyperplasia without platelet formation, an increase in eosinophilic series, and normal erythroid and myeloid series. Diagnosed with ITP and hemolytic anemia secondary to hepatitis A, the patient was treated with intravenous immunoglobulin 1gr/kg/day for two consecutive days. Three days after the start of IVIG, platelet count increased to 127000/mm^3^. As a result, she was discharged in a good condition. After 10 days, the hemoglobin level was within the normal range. Serial monitoring of CBC and liver function tests after discharge are shown in Table 1.

**Table 1:** Laboratory findings throughout the course of follow-up

(g/dl) Days	HB (/mm^3^)	WBC (/mm^3^)	PLT (IU/L)	SGOT (IU/L)	SGPT (IU/L)	ALP (mg/dl)	T. Bil (mg/dl)	D. Bil
Onset Day 3 Day 10 Day 30	8.6 9.2 11.2 11.5	14200 12700 7200 8900	14000 127000 185000 235000	602 483 93 49	617 524 174 35	1814 1580 837 617	3.5 3.3 1.2 0.6	1.8 1.7 0.3 0.2

## Discussion

 Hepatitis A is a common health problem in developing countries and usually with a mild or asymptomatic course in children. Jaundice occurs in about 5% of children under 3 years of age. 

Extrahepatic manifestations can appear in various organs which seem to be of immunological origin.^5^^, ^^6^ Also, ITP in children is often self-limiting and generally follows a viral infection, immunization, or inappropriate immune response.^7^ In a study, 14% of patients with HAV infection had atypical presentations such as cholestasis, acute hepatic failure, relapse, hematologic problems, and ascites.^8^ ITP and hemolytic anemia following hepatitis A have infrequently been reported as the initial manifestation or a symptom in later stages of disease. However, the simultaneous occurrence of them as the initial presentation has never been expressed. Autoimmune hemolytic anemia, secondary to hepatitis A has been alleged in an adult man by Tibble JA et al.^3^Tanir G. et al. also reported ITP as the only manifestation of HAV without jaundice in a 5-year-old boy whose platelets had become normal in two weeks after IVIG administration.^9^ In another study, Leblebısatan G. et al. presented cases of ITP secondary to hepatitis A in 2 children, 8 and 4 years old, who had respectively responded to IVIG and high-dose steroids.^10^ It seems that immune thrombocytopenia after hepatitis A results from generalized dysregulation of the immune system. Although ITP and hemolytic anemia after hepatitis A are really scarce, they are seen in developing countries due to a high prevalence of hepatitis A. Response to IVIG in our patient was rapid and significant, anemia and thrombocytopenia improved in a short period of time and in the last outpatient visit of patient, her clinical and laboratory studies were normal.

## CONCLUSION

 Most of patients with HAV infection present a classic form of disease. However, rare extrahepatic and hematological manifestations develop in a few patients. Immune thrombocytopenic purpura and hemolytic anemia may be seen rarely during the course of HAV infection in children. Hepatitis A virus infection should be among the differential diagnosis in any child presenting with ITP, especially in developing countries.
